# Adverse exposure burden, NMR metabolomics, and risk of incident abdominal aortic aneurysm

**DOI:** 10.3389/fpubh.2026.1842705

**Published:** 2026-07-23

**Authors:** Yi Yang, Xinyi Liu, Chengnan Li, Yipeng Ge, Haiou Hu, Zhiyu Qiao, Junming Zhu

**Affiliations:** Department of Cardiovascular Surgery, Beijing Aortic Disease Center, Beijing Anzhen Hospital of Capital Medical University, Beijing, China

**Keywords:** abdominal aortic aneurysm, mediation analysis, multidomain exposure burden, NMR metabolomics, risk prediction

## Abstract

**Background:**

The cumulative impact of multidomain adverse exposures on abdominal aortic aneurysm (AAA) risk and the underlying metabolic pathways remain insufficiently understood.

**Methods:**

We analyzed 304,482 UK Biobank participants free of aortic aneurysm at baseline. Twenty-six exposures were grouped into five domains to derive domain specific and overall exposure scores. Associations with incident AAA were assessed using Cox models. To explore potential metabolic pathways, we applied a two stage NMR metabolomics framework combining multivariable linear regression, metabolite specific Cox models, and a 10-fold cross validated elastic net Cox model to generate a metabolite score. Mediation analyses and XGBoost models were also performed.

**Results:**

During a mean follow up of 14.9 years, 1,671 participants developed AAA. A higher overall exposure score was associated with an increased risk of incident AAA (per SD increase: HR, 1.08; 95% CI, 1.07–1.11). Among domain specific scores, the socioeconomic, social psychology, and lifestyle scores were independently associated with AAA, whereas the environmental pollution and living environment scores were not. Current smoking showed the strongest association among individual exposures (HR, 7.95; 95% CI, 6.81–9.27). Overall exposure burden was associated with broad metabolomic perturbations, and a 13-metabolite score was significantly associated with AAA (per SD increase: HR, 1.38; 95% CI, 1.35–1.41), mediating 5.78% of this association. Adding exposure and metabolite scores improved prediction beyond clinical factors alone.

**Conclusions:**

Greater multidomain adverse exposure burden was associated with higher incident AAA risk, partly through metabolic signatures related to inflammation and lipoprotein metabolism, and may provide incremental value for AAA risk stratification.

## Introduction

Abdominal aortic aneurysm (AAA) remains a clinically important vascular disease because it often progresses silently before rupture, while current management still relies primarily on screening, surveillance, cardiovascular risk reduction, and timely repair rather than on effective disease modifying pharmacological therapies (1). In contemporary epidemiological research on AAA, increasing attention has been directed toward more refined risk stratified approaches to case identification. Most existing prediction models regard age, sex, smoking, body size, hypertension, dyslipidemia, and a history of cardiovascular disease as the principal determinants of risk (2, 3), yet substantial heterogeneity in susceptibility remains unexplained.

Moreover, AAA is unlikely to arise from isolated risk factors alone (4). Emerging cardiovascular research increasingly supports an exposome perspective, emphasizing that environmental, socioeconomic, psychosocial, and behavioral factors tend to cluster in real life and may jointly contribute to residual vascular risk beyond traditional clinical predictors (5, 6). In AAA, socioeconomic disadvantage has been associated with more severe clinical presentations, including aneurysm rupture (7), while unhealthy lifestyle patterns have also shown joint associations with aneurysm incidence in large population based cohorts (8). However, most AAA studies still focus on single exposures or a limited set of conventional predictors and therefore fail to fully capture the cumulative impact of multidomain exposure burden.

Metabolomics provides a highly promising intermediate layer linking upstream exposures to downstream aneurysm biology (9). Recent biomarker studies have shown that amino acid, inflammatory, and lipid related signatures are associated with AAA size, growth, or progression (10). However, most previous biomarker studies have been conducted in relatively small clinical cohorts and have rarely been integrated with multidomain exposure assessment in prospective population based studies. Therefore, it remains unclear whether exposure related metabolomic signatures mediate the association between cumulative exposure burden and incident AAA, and whether such information can improve risk prediction beyond clinical characteristics alone.

In summary, the present study aimed to quantify multidomain exposure burden across environmental pollution, living environment, socioeconomic, social psychology, and lifestyle domains; examine its association with incident AAA in the UK Biobank; identify exposure related NMR metabolomic signatures and their potential mediating roles; and evaluate whether integrating exposure and metabolite scores could improve AAA risk prediction.

## Methods

### Study design and participants

Data were derived from the UK Biobank, a nationwide prospective cohort including over 500,000 adults recruited throughout the United Kingdom from 2006 to 2010. Baseline information was collected according to standardized protocols, as described previously (11). Ethical approval for the UK Biobank was granted by the North West Multi-centre Research Ethics Committee (11/NW/0382), and written informed consent was obtained from all participants. Participants with a prior diagnosis of aortic aneurysm were excluded from this analysis, as were those with missing data on any exposure, covariate, NMR metabolomics measure, or outcome ascertainment. The final analytical cohort comprised 304,482 participants (Supplementary Figure S1).

### Exposure assessment

Exposure variables were grouped into five domains: environmental pollution, including NOx, NO_2_, PM2.5, PM10, PM2.5–10, and noise pollution; living environment, including greenspace and bluespace coverage; socioeconomic status, including household income, employment status, educational attainment, the index of multiple deprivation (IMD), and housing stability; social psychology factors, including social support, social activity, social isolation, living alone, loneliness, emotional distress, and psychiatric disorders; and lifestyle factors, including smoking status, alcohol consumption, physical activity, sedentary behavior, sleep quality, and diet. Detailed definitions for all exposures are presented in Supplementary Tables S1, S2.

To quantify the overall burden of exposure across heterogeneous indicators, we constructed domain-specific scores for each exposure domain according to the variable definitions shown in Supplementary Table S1. The environmental pollution score ranged from 0 to 6, the living environment score from 0 to 2, the socioeconomic score from 0 to 5, the social psychology score from 0 to 7, and the lifestyle score from 0 to 7. These five domain-specific scores were then summed to generate an overall exposure score ranging from 0 to 27. For all scores, a higher score indicated a more unfavorable composite exposure burden. Finally, each score was categorized into tertiles according to its distribution, with the lowest tertile representing a favorable exposure burden and the highest tertile representing an unfavorable exposure burden.

### Metabolomics measurements

Plasma metabolites in the UK Biobank were measured using a nuclear magnetic resonance (NMR)-based metabolomics platform. Procedures for blood sample collection, processing, and storage have been reported previously. EDTA plasma samples were profiled with the Nightingale Health NMR biomarker platform, which quantifies 251 metabolites, including 168 absolute concentrations and 83 ratios. For the present analysis, we retained the 168 metabolites measured as absolute concentrations and classified them into nine groups: amino acids, apolipoproteins, cholesterol, fatty acids, fluid balance, glycolysis-related metabolites, inflammation, ketone bodies, and lipoproteins and lipids. A complete list of metabolites is shown in Supplementary Table S3. Raw metabolite data were screened according to the quality control information provided by the UK Biobank NMR metabolomics platform. Outliers were defined as values below the median minus four times the interquartile range or above the median plus four times the interquartile range. Metabolite values that failed platform-specific quality control, had missing measurements, or were identified as outliers were excluded from metabolite-specific analyses. The remaining metabolite values were subsequently natural log-transformed and standardized as *z* scores prior to analysis.

### Covariate assessment

Covariates included age, sex, self-reported race, and body mass index (BMI); baseline history of diabetes, hypertension, dyslipidemia, chronic respiratory disease, chronic kidney disease, and cardiovascular disease; as well as prior use of lipid lowering, glucose lowering, and antihypertensive medications. Detailed definitions of medication use are provided in Supplementary Table S4.

### Outcomes

The outcome of this study was incident AAA, defined according to the International Classification of Diseases, Tenth Revision (ICD-10) codes I71.3 and I71.4. Cases of AAA were identified through death registry records, primary care data, hospital inpatient records, and self-reported diagnoses. The date of AAA onset was defined as the earliest recorded date of AAA diagnosis. Follow up was censored on August 31, 2024. Follow up time was calculated from the baseline assessment (2006 to 2010) to the date of AAA diagnosis, death, loss to follow up, or the end of follow up, whichever occurred first.

### Statistical analysis

Baseline characteristics were summarized as means (SDs) for continuous variables and counts (%) for categorical variables, with between group differences compared using one way analysis of variance or the chi-square test, as appropriate. Associations of 26 individual exposures, five domain specific exposure scores, and the overall exposure score with incident AAA were evaluated using Cox proportional hazards models, with hazard ratios (HRs) and 95% confidence intervals (CIs) estimated. The proportional hazards assumption was assessed using Schoenfeld residuals for the main Cox regression models. We fitted 26 single exposure models, one joint model including all individual exposures, five domain specific score models, one multivariable model including all domain specific scores, and a model for the overall exposure score. Bonferroni correction was applied in the single exposure analyses, and restricted cubic spline analyses were used to characterize dose response relationships for domain specific scores significantly associated with AAA. All models were adjusted for demographic factors, body mass index, baseline comorbidities, and medication use. Stratified analyses were performed by age, sex, and BMI category, and sensitivity analyses included excluding events occurring within the first 2 years of follow up, applying Fine-Gray competing-risk models, and excluding participants with baseline comorbidities. To explore potential metabolic pathways linking the overall exposure score to AAA, we used a two stage NMR metabolomics framework: multivariable linear regression was first used to identify metabolites associated with the overall exposure score, and Cox models were then used to identify metabolites associated with incident AAA, with Bonferroni correction applied at both stages. Metabolites significant in both analyses were entered into a 10-fold cross validated elastic net penalized Cox model to derive a weighted metabolite score, which was standardized as a *z* score. Mediation analyses were further performed to quantify the indirect effects of the metabolite score and selected metabolites. Finally, the cohort was randomly split into training (70%) and validation (30%) sets to develop XGBoost models for AAA prediction, with model performance assessed by discrimination, classification metrics, and decision curve analysis. Additional methodological details are provided in the Supplementary Methods.

## Results

### Baseline characteristics of participants

Baseline characteristics of the participants are presented in Table 1 and Supplementary Table S5. A total of 304,482 participants were included, with a mean age of 56.2 years (SD, 8.1 years), and 48.0% were male. During follow-up, 1,671 participants developed AAA. Compared with those who did not develop AAA, participants who developed AAA were more likely to be older, male, and overweight, and to have a greater burden of prior comorbidities and medication use. With respect to individual exposures, participants who developed AAA generally had more adverse socioeconomic conditions, more frequent loneliness and social isolation, and less healthy lifestyle habits. However, no significant differences were observed in overall environmental pollution or living environment factors. To assess potential selection bias related to the complete-case analysis, we compared baseline characteristics between included and excluded participants, and the overall differences were generally modest (Supplementary Table S6).

**Table 1:** Baseline characteristics of participants.

Characteristics	Non-AAA	Incident AAA	*p*-value
*N*	302,811	1,671	
Age, years (SD)	56.1 ± 8.07	63.2 ± 5.03	< 0.001
Male (%)	144,789 (47.8%)	1,419 (84.9%)	< 0.001
White ethnicity or race (%)	290,590 (96.0%)	1,642 (98.3%)	< 0.001
BMI (SD)	27.2 ± 4.54	28.5 ± 4.26	< 0.001
Medical history			
Hypertension (%)	89,474 (29.5%)	949 (56.8%)	< 0.001
Diabetes (%)	22,127 (7.31%)	244 (14.6%)	< 0.001
Dyslipidemia (%)	114,112 (37.7%)	893 (53.4%)	< 0.001
Chronic respiratory diseases (%)	39,866 (13.2%)	264 (15.8%)	0.002
Chronic kidney disease (%)	1,049 (0.35%)	17 (1.02%)	< 0.001
Cardiovascular disease (%)	30,380 (10.0%)	609 (36.4%)	< 0.001
Lipid-lowering medication (%)	44,330 (14.6%)	716 (42.8%)	< 0.001
Antihypertensive medication (%)	60,184 (19.9%)	793 (47.5%)	< 0.001
Antidiabetic medication (%)	9,715 (3.2%)	90 (5.4%)	< 0.001

p-Values were determined using the ANOVA test for continuous variables and the chi-square test for categorical variables. BMI, body mass index.

### Associations between individual exposure factors and the risk of incident AAA

**Figure 1:**
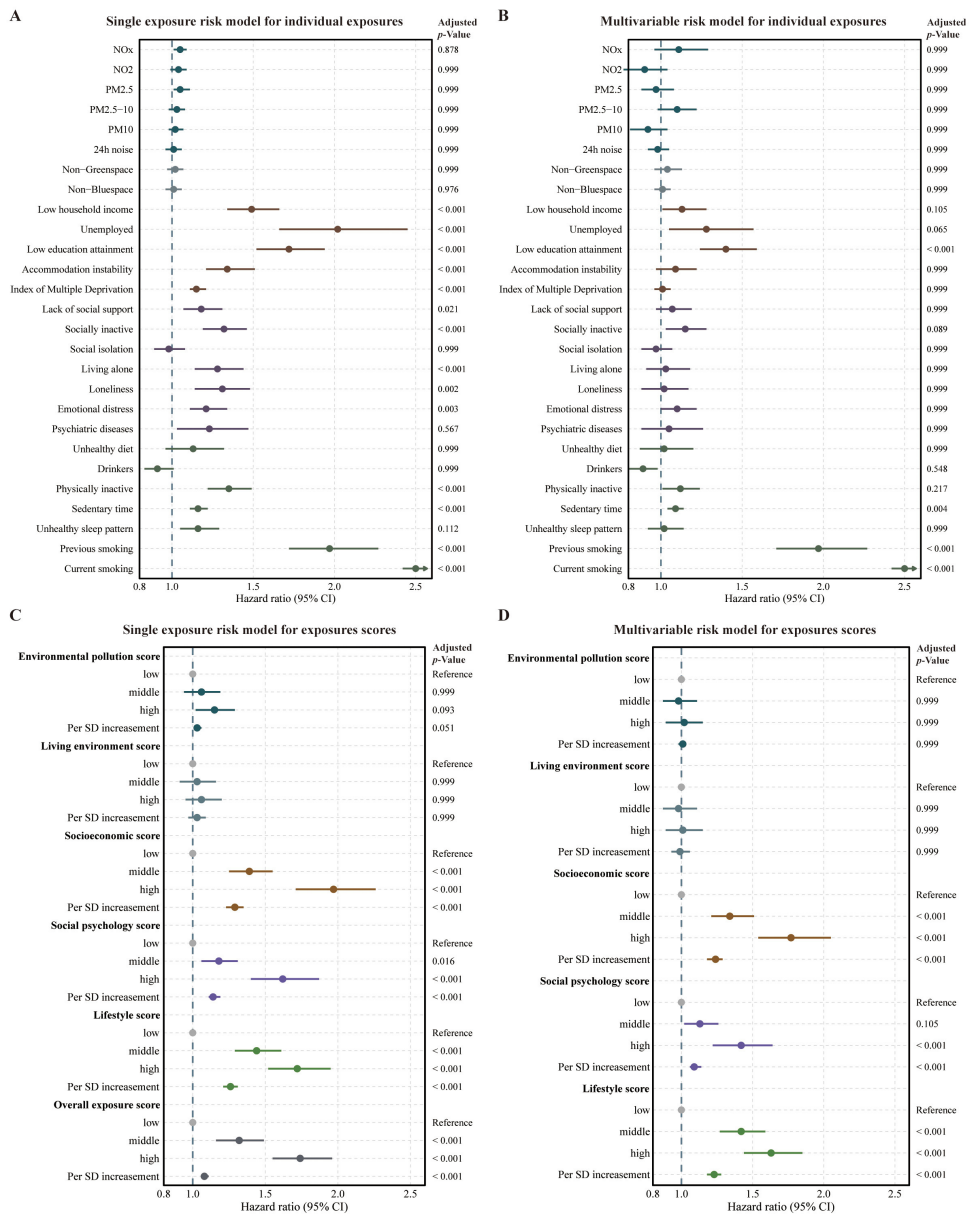
Associations of exposure factors and exposure scores with the risk of incident AAA. **(A, B)** Single exposure models and a joint exposure model based on Cox proportional hazards regression were used to evaluate the associations between individual exposures and the risk of incident AAA. **(C, D)** Cox proportional hazards regression models were used to assess the associations between multidomain exposure scores and the risk of incident AAA. All models were adjusted for sex, age, self-reported race, BMI, baseline comorbidities (hypertension, diabetes, dyslipidemia, chronic respiratory disease, chronic kidney disease, and cardiovascular disease), and prior use of antihypertensive, antidiabetic, and lipid-lowering medications. *P* values were Bonferroni adjusted.

Over a mean follow up of 14.9 years, we observed varying associations between individual exposures and the risk of incident AAA, as shown in Figure 1A. Notably, current smoking showed the strongest association with AAA risk. Compared with never smoking, current smoking was associated with a markedly increased risk of AAA (HR, 7.95; 95% CI, 6.81–9.27). Employment status, educational attainment, and several lifestyle related factors also showed associations of varying magnitudes. In the multiple exposure model (Figure 1B), these exposures showed generally similar effects, whereas no significant association was observed between air pollution related exposures and the risk of incident AAA. Given the potential nonlinearity among exposures, we further constructed domain specific exposure scores across the five domains, along with an overall exposure score.

As shown in Supplementary Figure S2, compared with the lowest tertile of the overall exposure score, the highest tertile was associated with a substantially higher incidence of AAA, reaching approximately 0.468 cases per 1,000 person years. Among the domain specific scores, the highest tertile of the lifestyle score showed the greatest incidence of AAA, at approximately 0.604 cases per 1,000 person years. As shown in Figure 1C, each 1 SD increase in the overall exposure score was associated with an approximately 8% higher risk of incident AAA (per SD increase: HR, 1.08; 95% CI, 1.07–1.11; tertile 3 vs. tertile 1: HR, 1.74; 95% CI, 1.55–1.96). Among the domain specific scores, the socioeconomic score, social psychology score, and lifestyle score were all significantly associated with AAA risk. In contrast, no association was observed for the environmental pollution score or the living environment score. As shown in Figure 1D, in the multivariable model including all domain specific scores, all domains except environmental pollution and living environment remained significantly associated with the risk of incident AAA.

**Figure 2:**
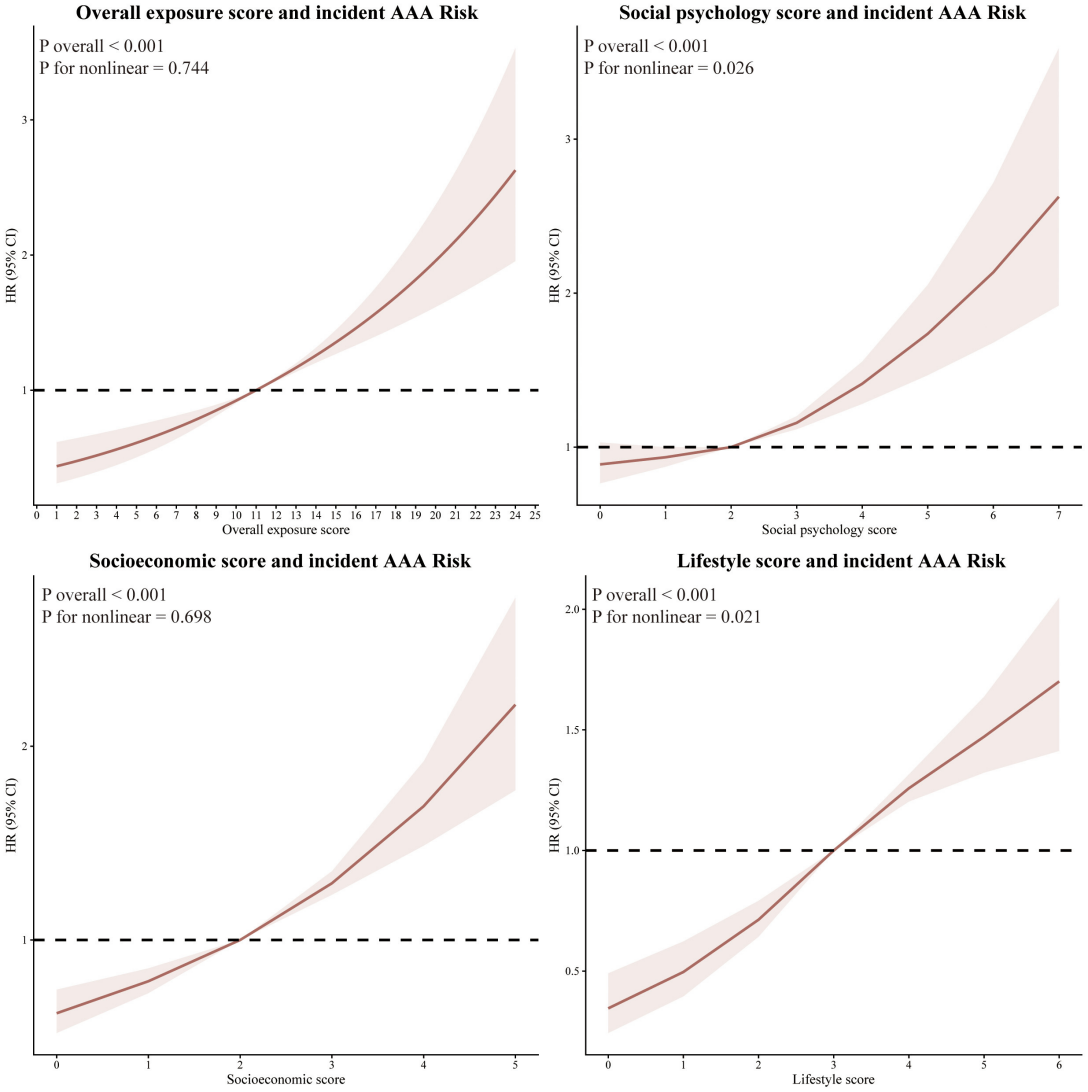
Restricted cubic spline analyses of the associations between domain specific exposure scores and the risk of incident AAA. All analyses were adjusted for sex, age, self-reported race, BMI, baseline comorbidities (hypertension, diabetes, dyslipidemia, chronic respiratory disease, chronic kidney disease, and cardiovascular disease), and prior use of antihypertensive, antidiabetic, and lipid-lowering medications. *P* values were Bonferroni adjusted.

We next used restricted cubic splines to characterize the dose response associations between domain specific exposure scores and AAA risk. As shown in Figure 2, no evidence of nonlinearity was observed for the association between the overall exposure score and AAA risk (*P* for nonlinearity = 0.744). However, significant nonlinear associations were identified for the social psychology score and the lifestyle score (both *P* for nonlinearity < 0.05). As shown in Supplementary Table S7, the associations between individual exposure scores and AAA risk were generally consistent across subgroups. We also performed multiple sensitivity analyses (Supplementary Table S8), including exclusion of events occurring within the first 2 years of follow up, application of competing risk models, and exclusion of participants with major baseline comorbidities. The results remained consistent with those of the primary analyses.

### Associations of exposure scores and related metabolites with the risk of incident AAA

**Figure 3:**
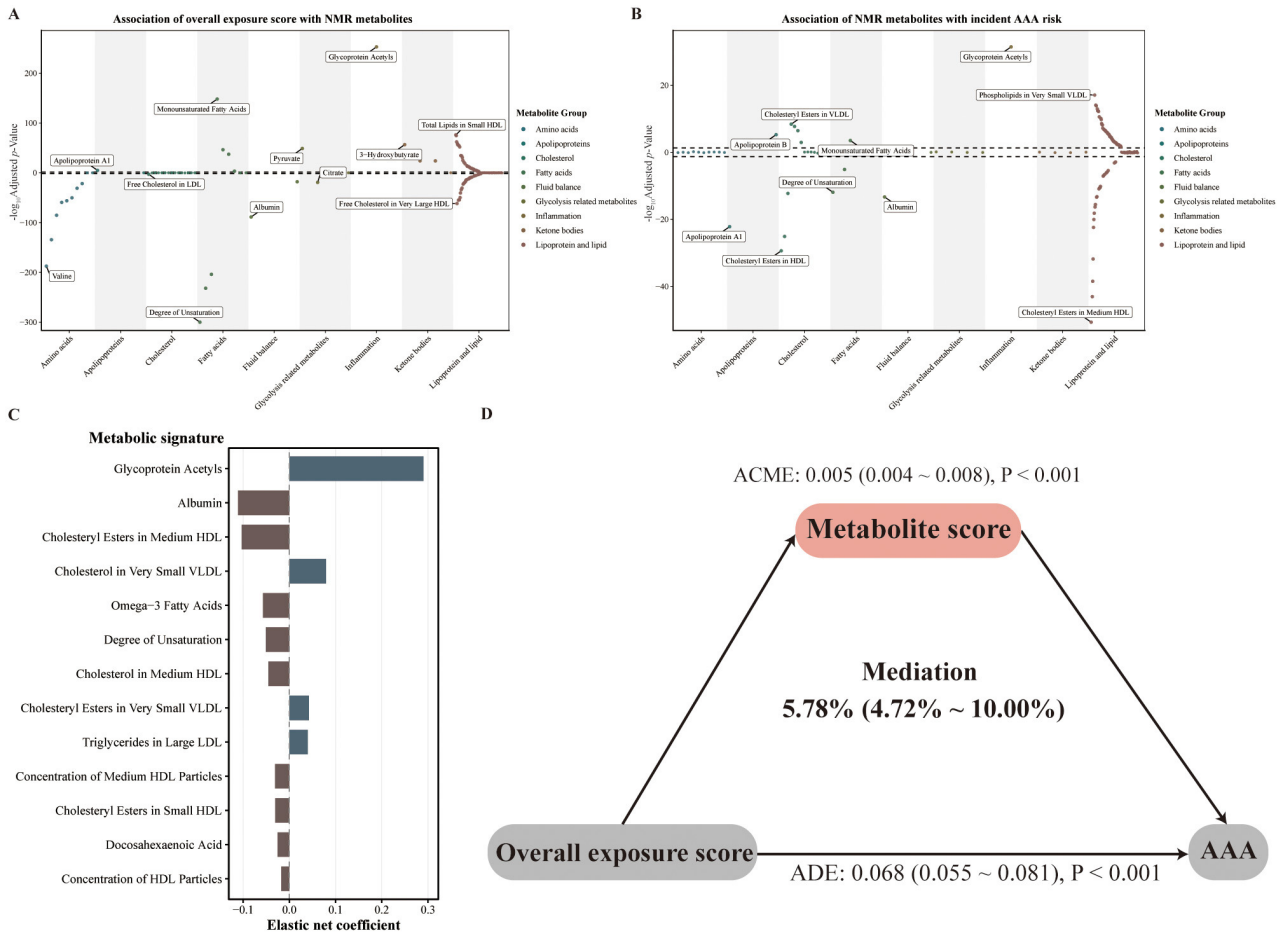
Two stage NMR metabolomics analysis of the association between the overall exposure score and the risk of incident AAA. **(A)** Association of the overall exposure score with the NMR metabolomic profile. **(B)** Association of NMR metabolites with the risk of incident AAA. **(C)** Key metabolites with nonzero coefficients. **(D)** Potential mediating role of the metabolite score in the association between the overall exposure score and the risk of incident AAA. In both stages, analyses were adjusted for sex, age, self-reported race, BMI, baseline comorbidities (hypertension, diabetes, dyslipidemia, chronic respiratory disease, chronic kidney disease, and cardiovascular disease), and prior use of antihypertensive, antidiabetic, and lipid-lowering medications. ACME, average causal mediation effect; ADE, average direct effect.

We further evaluated the associations between NMR metabolomics and multidomain exposure scores. As shown in Figure 3A, among 168 metabolites quantified as absolute concentrations, 92 were significantly associated with the overall exposure score after Bonferroni correction, with most belonging to lipoprotein and lipid related measures. In the domain specific analyses (Supplementary Figure S3), we focused on the three domain scores other than environmental pollution and living environment, because neither of these two domains showed a clear association with incident AAA in the earlier analyses. The remaining three domain scores showed broadly similar association patterns with the metabolomic profile. As shown in Figure 3B, the prospective analysis demonstrated that 93 metabolites were significantly associated with incident AAA in the fully adjusted Cox models, again driven predominantly by lipoprotein and lipid related measures. After integrating the significant metabolites identified in the two stages, the number of overlapping AAA related metabolites differed across domain specific scores, and the shared signals were mainly driven by lipoproteins, lipids, and amino acids, with the lifestyle score showing the greatest overlap (Supplementary Figure S3D).

Given the potential correlations and collinearity across domains, subsequent analyses focused on the overall exposure score. We then applied an elastic net penalized Cox proportional hazards model to candidate metabolites that were significant in both stages and were associated with both the overall exposure score and AAA risk. As shown in Figure 3C, 13 key metabolites with nonzero coefficients were retained. In addition, the correlation structure shown in Supplementary Figure S4 indicated that these AAA related metabolites were not confined to a single highly correlated cluster. We subsequently constructed a metabolite score on the basis of the concentrations and coefficients of these 13 key metabolites. As shown in Supplementary Table S9, each 1 SD increase in the metabolite score was associated with an approximately 38% higher risk of incident AAA (HR, 1.38; 95% CI, 1.35–1.41). As shown in Supplementary Figure S5, the metabolite score showed a significant nonlinear association with AAA risk (*P* for nonlinearity < 0.001). We further explored the potential mediating roles of individual metabolites and the metabolite score. As shown in Figure 3D, the metabolite score significantly mediated the association between the overall exposure score and incident AAA, accounting for approximately 5.78% of the total effect (95% CI, 4.72%−10.00%). As shown in Supplementary Table S10, among the 13 key metabolites, Glycoprotein Acetyls showed a positive mediation proportion of approximately 6.19% (95% CI, 4.97%−8.22%). In contrast, Cholesteryl Esters in Small HDL showed the largest negative mediation proportion, at approximately −2.47% (95% CI, −3.26% to −1.89%).

**Figure 4:**
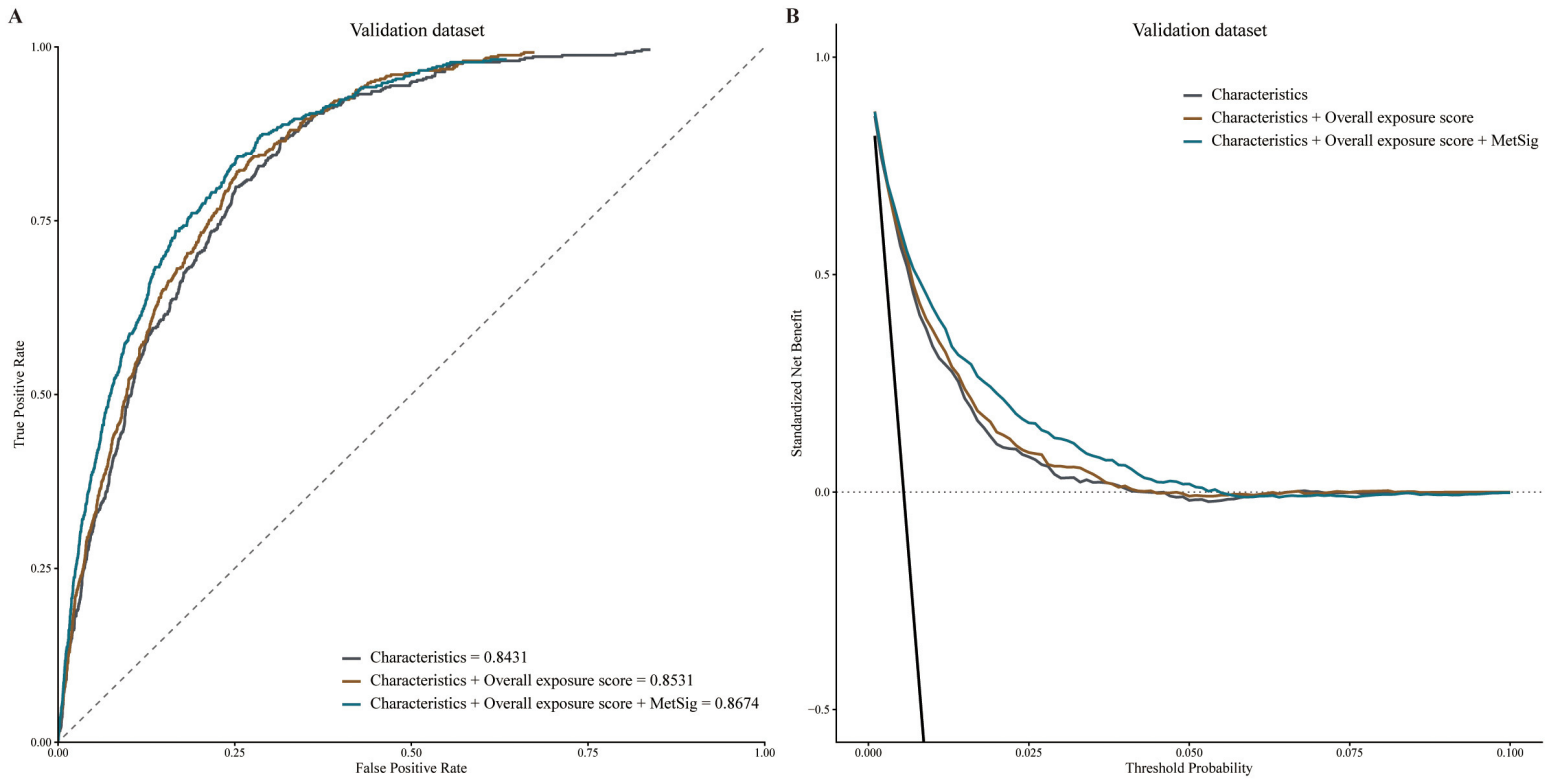
Machine learning models for AAA prediction. **(A)** Receiver operating characteristic curves. **(B)** Decision curve analysis. Characteristics included sex, age, self-reported race, BMI, baseline comorbidities (hypertension, diabetes, dyslipidemia, chronic respiratory disease, chronic kidney disease, and cardiovascular disease), and prior use of antihypertensive, antidiabetic, and lipid-lowering medications.

We finally evaluated the potential of the overall exposure score and metabolite score for predicting AAA events. Using XGBoost, we developed three risk prediction models for AAA. All models were trained in 70% of the cohort and validated in the remaining 30%. As shown in Figure 4A, compared with Model 1, which included clinical covariates alone (AUC = 0.8431), the addition of the overall exposure score in Model 2 slightly improved predictive performance (AUC = 0.8531). After further incorporating the metabolite score, Model 3 showed additional improvement (AUC = 0.8674). In addition, decision curve analysis (Figure 4B) showed that Model 3 provided the greatest net clinical benefit.

## Discussion

In this large prospective cohort study, we found that the overall exposure score was independently associated with an increased risk of incident AAA. Among individual exposures, smoking showed the strongest association with AAA, whereas at the domain level, the socioeconomic, social psychology, and lifestyle scores were significantly associated with AAA risk, while the environmental pollution and living environment scores were not. We further demonstrated that the overall exposure burden was associated with widespread perturbations in the NMR metabolomic profile, with overlapping metabolites related to both exposure and AAA, mainly involving lipoprotein, lipid, and amino acid measures. A metabolite score incorporating 13 metabolites was strongly associated with incident AAA, partially mediated the association between the overall exposure score and AAA, and modestly improved risk prediction beyond traditional clinical characteristics. Taken together, our findings suggest that the association between adverse multidomain exposure burden and incident AAA may be partly reflected in circulating metabolic signatures related to systemic inflammation and lipoprotein metabolism.

Our findings suggest that lifestyle, socioeconomic, and psychosocial factors, rather than environmental pollution or living environment, constitute the major components of exposure burden associated with AAA. Among these, smoking remained the predominant exposure factor for AAA, further underscoring the central role of smoking related vascular injury in aneurysm formation (4). Previous studies have likewise shown that unhealthy lifestyle patterns are closely associated with incident AAA, with smoking representing the largest contributor to the excess risk (8). At the same time, the independent association of socioeconomic burden observed in our study is consistent with recent reports linking poverty to aneurysm rupture and adverse outcomes (7). Taken together, these data support the view that AAA should not be regarded merely as a disease related to aging and smoking, but rather as a condition embedded within broader behavioral and social vulnerability. In contrast, our null findings for environmental pollution do not necessarily exclude a role for environmental exposures. A recent large prospective study from the UK Biobank reported a significant positive association between long term exposure to air pollutants and the incidence of AAA (12), suggesting that, within our score based and jointly adjusted framework, the dominant signal may have shifted toward more proximal behavioral and socioeconomic determinants. This discrepancy may be partly explained by differences in exposure modeling and covariate adjustment. Unlike previous studies that mainly examined individual air pollutants, our study used a harmonized domain-specific pollution score and simultaneously adjusted for socioeconomic, psychosocial, lifestyle, clinical, and medication-related factors, which may have attenuated the independent association of environmental pollution with AAA. Therefore, our null finding for the environmental pollution score should not be interpreted as excluding a possible role of air pollution in AAA.

Our metabolomics analysis provides further biological context for these epidemiological associations and suggests that lipoprotein and inflammatory pathways may represent key links between cumulative exposure burden and AAA risk. Most metabolites associated with both the overall exposure score and incident AAA belonged to lipoprotein and lipid related measures, and the 13 key metabolite features were not confined to a single highly correlated cluster, suggesting that these findings do not simply reflect redundant lipid signals. This pattern is consistent with recent biomarker studies showing that circulating metabolomic signatures are associated with AAA burden and progression (9, 13). Of particular note in our analysis was the positive mediating effect of Glycoprotein Acetyls. Glycoprotein Acetyls is an NMR derived marker of chronic systemic inflammation and has increasingly been recognized as a robust signal of cardiovascular risk (14, 15). In addition, Cholesteryl Esters in Small HDL showed a negative mediating effect. This observation is directionally consistent with emerging prospective evidence suggesting that lower HDL cholesterol levels are associated with a higher risk of incident AAA (16). Taken together, our data support a model in which adverse multidomain exposures may partly promote AAA development through metabolic inflammation and disturbed lipoprotein metabolism, although the modest mediation proportion suggests that other important biological pathways beyond the measured metabolome are also likely to contribute.

From a clinical prediction perspective, the stepwise improvement in discrimination and decision curve performance after adding the overall exposure score and metabolite score suggests that integrated exposure based and molecular profiling approaches may help refine conventional AAA risk stratification. Current AAA risk models and targeted screening strategies still rely primarily on age, sex, smoking related variables, and traditional clinical history (2, 3). Although these factors are clinically useful, they do not fully capture the heterogeneity of susceptibility. Our findings indicate that multidomain exposure burden provides additional prognostic information beyond standard clinical covariates, and that this information can be further refined by a parsimonious metabolite score. This is consistent with recent large scale cardiovascular studies showing that the addition of NMR derived metabolomic scores to established clinical models can improve risk prediction (15). At the same time, the gain in AUC observed in the present study was modest. Accordingly, these markers should be regarded mainly as complementary tools for risk enrichment and biological phenotyping, rather than as complete replacements for existing screening and surveillance strategies. In addition, the clinical value of the metabolite score may lie particularly in identifying individuals whose risk is underestimated by conventional assessment and in supporting a more prevention oriented approach to AAA risk evaluation. However, before clinical implementation, further calibration assessment, and cost-effectiveness analyses are needed to determine whether the incremental predictive gain is sufficient to support routine use.

Our study has several strengths. First, using a large prospective cohort, we constructed five domain specific scores together with an overall exposure score to evaluate their associations with the risk of AAA events. Second, we integrated large scale NMR metabolomics data to explore potential biological pathways linking the overall exposure score to AAA. Several limitations should also be acknowledged. First, although we adjusted for a wide range of potential confounders, residual confounding from unmeasured factors cannot be fully excluded. Second, mechanistic inferences were based primarily on association analyses of metabolomics data, and further functional studies are needed for validation. Third, the lack of detailed information on AAA size in the UK Biobank prevented us from incorporating aneurysm diameter into the analysis. Fourth, because most participants in the UK Biobank were White adults, the generalizability of our findings to other racial and ethnic groups may be limited. Finally, the predictive value of the overall exposure score and metabolite score for AAA risk still requires validation in additional external cohorts.

## Conclusions

In conclusion, a higher overall exposure burden was closely associated with an increased risk of incident AAA, and this association was driven mainly by socioeconomic, social psychological, and lifestyle factors. In addition, a metabolite score derived from 13 key metabolites identified through NMR metabolomics was significantly associated with AAA and mediated approximately 5.78% of the association between overall exposure burden and AAA risk, with inflammation related and lipoprotein related molecular phenotypes potentially contributing to this biological mediation. In terms of risk prediction, adding the exposure score and metabolite score to models containing conventional clinical factors provided a modest improvement in predictive performance. Together, these findings may help refine AAA risk stratification and prevention, while also highlighting potentially modifiable upstream pathways for future investigation.

## Data Availability

The original contributions presented in the study are included in the article/Supplementary material, further inquiries can be directed to the corresponding author.
