# Supplementary material for: Adverse exposure burden, NMR metabolomics, and risk of incident abdominal aortic aneurysm
Source: Front Public Health. 2026 Jul 23;14:1842705. doi: 10.3389/fpubh.2026.1842705 (PMC13443055; doi:10.3389/fpubh.2026.1842705)
Supplement: Supplementary file 1 [file Data_Sheet_1.docx]

**Supplementary Material**

**Supplementary Methods**

**Supplementary Tables**

**Supplementary Table 1.** Detailed definitions of exposures.

**Supplementary Table 2.** Components of an ideal diet.

**Supplementary Table 3.** Summary statistics of plasma metabolites.

**Supplementary Table 4.** Drug classification and data coding.

**Supplementary Table 5.** Baseline characteristics of individual exposures among participants.

**Supplementary Table 6.** Baseline characteristics of included and excluded participants.

**Supplementary Table 7.** Subgroup analyses of the associations between multidomain exposure scores and the risk of incident AAA.

**Supplementary Table 8.** Sensitivity analyses of the associations between multidomain exposure scores and the risk of incident AAA.

**Supplementary Table 9.** Association between the metabolite score and the risk of incident AAA.

**Supplementary Table 10.** Mediating effects of key metabolites in the association between the overall exposure score and the risk of incident AAA.

**Supplementary Figures**

**Supplementary Figure 1.** Study flowchart.

**Supplementary Figure 2.** Incidence of AAA according to categories of multidomain exposure scores.

**Supplementary Table 3.** Associations between domain specific exposure scores and the NMR metabolomic profile.

**Supplementary Figure 4.** Spearman correlation heatmap of metabolites included in the elastic net cox model.

**Supplementary Figure 5.** Restricted cubic spline analysis of the association between the metabolite score and the risk of incident AAA.

**Supplementary Methods**

**Statistical analysis**

Baseline characteristics were summarized as means (SDs) for continuous variables and counts (percentages) for categorical variables, with group differences compared using one-way analysis of variance or the chi-square test, as appropriate. Cox proportional hazards regression models were used to estimate hazard ratios (HRs) and 95% confidence intervals (CIs). We constructed 26 single-exposure models and one joint model including all exposure factors to evaluate the associations between the 26 individual exposures and AAA. In addition, we constructed five exposure score models and one multivariable model including all exposure scores to assess the associations between each exposure score and AAA. Finally, we evaluated the association between the overall exposure score and incident AAA. In the single-exposure models, P values were adjusted using the Bonferroni method. Subsequently, restricted cubic spline (RCS) analyses were performed to examine the associations between domain-specific exposure scores that were significantly associated with AAA and the risk of incident AAA. All models were adjusted for sex, age, self-reported race, BMI, baseline medical history (hypertension, diabetes, dyslipidemia, chronic respiratory disease, chronic kidney disease, and cardiovascular disease), and prior medication use (antihypertensive, antidiabetic, and lipid-lowering medications) to reduce potential confounding. In addition, stratified analyses were conducted according to age, sex, and BMI category (normal BMI: 18.5 to 24.9; abnormal BMI), and a series of sensitivity analyses were performed to assess the robustness of the findings. First, to reduce potential reverse causation, we excluded events occurring within the first 2 years of follow-up. Second, Fine-Gray subdistribution hazard models were applied to account for the competing risk of non-AAA death and censoring due to loss to follow-up. Finally, to account for the potential influence of baseline comorbidities, we excluded participants with a prior medical history.

To further investigate potential pathways connecting the overall exposure score with incident AAA, we adopted a two-stage NMR metabolomics analytical framework. In the first stage, we used multivariable linear regression to examine the association between the overall exposure score and each metabolite, treating the metabolite as the dependent variable and adjusting for the covariates described above. In the second stage, we fitted Cox proportional hazards models for incident AAA for each metabolite using the same set of covariates. Bonferroni correction was applied in both the metabolite-wide linear regression analyses and the metabolite-specific Cox models to account for multiple comparisons. Metabolites that were significant in both stages were then entered into a 10-fold cross-validated elastic net penalized Cox proportional hazards model to identify the key metabolites related to AAA risk. This elastic net method integrates L1 (lasso) and L2 (ridge) penalties, enabling simultaneous variable selection and regularization by shrinking coefficients of less informative features toward zero while preserving relevant predictive metabolites. Thirteen metabolites with non-zero coefficients were ultimately retained and used to derive a metabolite score, which was calculated as the weighted sum of the selected metabolite concentrations, with weights defined by the coefficients from the elastic net Cox model. This score was subsequently standardized as a z score for further analyses. We also performed mediation analyses to assess whether the metabolite score and the 13 selected metabolites mediated the association between the overall exposure score and incident AAA. All mediation models were based on the fully adjusted covariate set. Indirect effects were estimated using quasi-Bayesian Monte Carlo simulations with 1,000 iterations, from which the average causal mediation effect (ACME), proportion mediated, and corresponding 95% confidence intervals were obtained.

Finally, to validate the utility of the overall exposure score and metabolite score, the entire cohort was randomly divided into a training set (70%) and a validation set (30%). Using extreme gradient boosting (XGBoost), we developed three prediction models for AAA. Model 1 included clinical characteristics and all covariates. Model 2 included the variables in Model 1 plus the overall exposure score. Model 3 further incorporated the metabolite score in addition to Model 2. Model performance was evaluated using the area under the receiver operating characteristic curve (AUC), accuracy, sensitivity, and specificity. Decision curve analysis was also performed to assess clinical utility across a range of risk thresholds.

All statistical analyses were conducted using R software (version 4.4.2; R Foundation for Statistical Computing). Two-sided tests were applied, and p-values <0.05 were considered statistically significant.

**Supplementary Table**

**Supplementary Table 1. Detailed definitions of exposures.**

| **Exposure** | **Field IDs** | **Definition** |
| --- | --- | --- |
| **Environmental pollution** | | |
| NOx, NO2, PM2.5, PM10, PM2.5-10 | 24004, 24003, 24006, 24005, 24008 | To ensure consistency in data harmonization, we used the 2010 concentrations of all pollutants as the exposure metrics. Annual mean air pollutant concentrations were estimated using land-use regression (LUR) models. Dichotomized by the median (0: < median; 1: ≥ median) |
| Noise pollution | 24024 | Based on the 24-hour average sound level for each residential address in 2010, which was modeled using a LUR approach. Dichotomized by the median (0: < median; 1: ≥ median) |
| **Living environment** | | |
| Residential greenspace | 24503 | Proportion of greenspace within a 300m buffer around the residential address; values were reverse-coded so that higher values indicate lower coverage. Dichotomized by the median (0: < median; 1: ≥ median) |
| Residential bluespace | 24505 | Proportion of water coverage within a 300m buffer around the residential address; values were reverse-coded so that higher values indicate lower coverage. Dichotomized by the median (0: < median; 1: ≥ median) |
| **Socioeconomic** | | |
| Household income | 738 | 0: average pre-tax total household income >£31,000;  1: <£31,000 |
| Employment status | 6142 | 0: employed, retired, or a student;  1: unemployed |
| Educational attainment | 6138 | 0: highest educational attainment at university level or above; 1: below university level |
| Index of Multiple Deprivation | 26410 | The 2010 UK Index of Multiple Deprivation (IMD) was used to assess area-level multiple deprivation. Dichotomized by the median (0: < median; 1: ≥ median) |
| Accommodation stability | 680 | 0: owned the home outright; 1: renting or other arrangements |
| **Social psychology** | | |
| Social support | 2110 | 0: at least once per week, confiding in any close person; 1: less than once per week |
| Social activity | 6160 | 0: participated in an activity at least once per week; 1: less than once per week |
| Social isolation | 1031 | 0: visited family or had them visit you at least once per week; 1: less than once per week |
| Living alone | 709 | 0: living with others; 1: living alone |
| Loneliness | 2020 | 0: none; 1: often felt lonely |
| Emotional distress | 6145 | 0: none; 1: experienced illness/injury, bereavement, or stress in the past two years |
| Psychiatric disorders | 41270 | 0: none; 1: previously diagnosed with a mental disorder |
| **Lifestyle** | | |
| Smoking status | 1239, 1249, 2644 | Categorized as never smokers (0), former smokers (1), and current smokers (2). |
| Alcohol intake | 1558, 1568, 1578, 1588, 1598, 1608, 5364 | 0: daily alcohol intake (men <2 drinks, women <1 drink); 1: daily alcohol intake (men ≥2 drinks, women ≥1 drink) |
| Physical activity | 971, 981, 991, 1001, 2624, 2634, 3637, 3647 | Based on weekly metabolic equivalent of task (MET) minutes. 0: <600 MET-min/week; 1: ≥600 MET-min/week |
| Sedentary behavior | 1070, 1080 | 0: less than the median sedentary time; 1: greater than or equal to the median sedentary time |
| Sleep quality | 1160, 1180, 1200, 1210, 1220 | For each component of the sleep pattern, the high-risk component was scored as 1 and the low-risk component as 0. 0: component score of 3-5; 1: score of 0-2 |
| Diet | Supplementary table 3 | 0: a diet including at least five major food components; 1: fewer than five |

**Supplementary Table 2. Components of an ideal diet.**

| **Diet component** | **Field IDs** | **Intake goal** |
| --- | --- | --- |
| Fruit | 1309, 1319 | ≥3 servings/day |
| Vegetable | 1289, 1299 | ≥3 servings/day |
| Whole grains | 1438/1448, 1458/1468 | ≥3 servings/day |
| Fish (Shell) | 1329, 1339 | ≥2 servings/day |
| Dairy | 1408, 1418 | ≥2 servings/day |
| Vegetable oils | 1428, 1438, 2654 | ≥2 servings/day |
| Refined grains | 1438/1448, 1458/1468 | ≤2 servings/day |
| processed meats | 1349, 3680 | ≤1 servings/day |
| Unprocessed meats | 1359, 1369, 1379, 1389, 3680 | ≤2 servings/day |
| Sugar-sweetened beverages | 6144 | No consumption |

**Supplementary Table 3. Summary statistics of plasma metabolites.**

| **Metabolite** | **Metabolite group** | **Unit** | **UKB field ID** | **QC Flag Field ID** |
| --- | --- | --- | --- | --- |
| Alanine | Amino acids | mmol/L | 23460 | 23760 |
| Glutamine | Amino acids | mmol/L | 23461 | 23761 |
| Glycine | Amino acids | mmol/L | 23462 | 23762 |
| Histidine | Amino acids | mmol/L | 23463 | 23763 |
| Isoleucine | Amino acids | mmol/L | 23465 | 23765 |
| Leucine | Amino acids | mmol/L | 23466 | 23766 |
| Phenylalanine | Amino acids | mmol/L | 23468 | 23768 |
| Total Concentration of Branched-Chain Amino Acids (Leucine + Isoleucine + Valine) | Amino acids | mmol/L | 23464 | 23764 |
| Tyrosine | Amino acids | mmol/L | 23469 | 23769 |
| Valine | Amino acids | mmol/L | 23467 | 23767 |
| Apolipoprotein A1 | Apolipoproteins | g/l | 23440 | 23740 |
| Apolipoprotein B | Apolipoproteins | g/l | 23439 | 23739 |
| Clinical LDL Cholesterol | Cholesterol | mmol/L | 23404 | 23704 |
| HDL Cholesterol | Cholesterol | mmol/L | 23406 | 23706 |
| Cholesteryl Esters in HDL | Cholesterol | mmol/L | 23418 | 23718 |
| Free Cholesterol in HDL | Cholesterol | mmol/L | 23422 | 23722 |
| LDL Cholesterol | Cholesterol | mmol/L | 23405 | 23705 |
| Cholesteryl Esters in LDL | Cholesterol | mmol/L | 23417 | 23717 |
| Free Cholesterol in LDL | Cholesterol | mmol/L | 23421 | 23721 |
| Total Cholesterol Minus HDL-C | Cholesterol | mmol/L | 23401 | 23701 |
| Remnant Cholesterol (Non-HDL, Non-LDL -Cholesterol) | Cholesterol | mmol/L | 23402 | 23702 |
| Total Cholesterol | Cholesterol | mmol/L | 23400 | 23700 |
| Total Esterified Cholesterol | Cholesterol | mmol/L | 23415 | 23715 |
| Total Free Cholesterol | Cholesterol | mmol/L | 23419 | 23719 |
| VLDL Cholesterol | Cholesterol | mmol/L | 23403 | 23703 |
| Cholesteryl Esters in VLDL | Cholesterol | mmol/L | 23416 | 23716 |
| Free Cholesterol in VLDL | Cholesterol | mmol/L | 23420 | 23720 |
| Docosahexaenoic Acid | Fatty acids | mmol/L | 23450 | 23750 |
| Linoleic Acid | Fatty acids | mmol/L | 23449 | 23749 |
| Monounsaturated Fatty Acids | Fatty acids | mmol/L | 23447 | 23747 |
| Omega-3 Fatty Acids | Fatty acids | mmol/L | 23444 | 23744 |
| Omega-6 Fatty Acids | Fatty acids | mmol/L | 23445 | 23745 |
| Polyunsaturated Fatty Acids | Fatty acids | mmol/L | 23446 | 23746 |
| Saturated Fatty Acids | Fatty acids | mmol/L | 23448 | 23748 |
| Total Fatty Acids | Fatty acids | mmol/L | 23442 | 23742 |
| Degree of Unsaturation | Fatty acids | degree | 23443 | 23743 |
| Albumin | Fluid balance | g/l | 23479 | 23779 |
| Creatinine | Fluid balance | mmol/L | 23478 | 23778 |
| Citrate | Glycolysis related metabolites | mmol/L | 23473 | 23773 |
| Glucose | Glycolysis related metabolites | mmol/L | 23470 | 23770 |
| Lactate | Glycolysis related metabolites | mmol/L | 23471 | 23771 |
| Pyruvate | Glycolysis related metabolites | mmol/L | 23472 | 23772 |
| Glycoprotein Acetyls | Inflammation | mmol/L | 23480 | 23780 |
| 3-Hydroxybutyrate | Ketone bodies | mmol/L | 23474 | 23774 |
| Acetate | Ketone bodies | mmol/L | 23475 | 23775 |
| Acetoacetate | Ketone bodies | mmol/L | 23476 | 23776 |
| Acetone | Ketone bodies | mmol/L | 23477 | 23777 |
| Average Diameter for HDL Particles | Lipoprotein and lipid | nm | 23433 | 23733 |
| Average Diameter for LDL Particles | Lipoprotein and lipid | nm | 23432 | 23732 |
| Average Diameter for VLDL Particles | Lipoprotein and lipid | nm | 23431 | 23731 |
| Cholesterol in Chylomicrons and Extremely Large VLDL | Lipoprotein and lipid | mmol/L | 23484 | 23784 |
| Cholesterol in IDL | Lipoprotein and lipid | mmol/L | 23526 | 23826 |
| Cholesterol in Large HDL | Lipoprotein and lipid | mmol/L | 23561 | 23861 |
| Cholesterol in Large LDL | Lipoprotein and lipid | mmol/L | 23533 | 23833 |
| Cholesterol in Large VLDL | Lipoprotein and lipid | mmol/L | 23498 | 23798 |
| Cholesterol in Medium HDL | Lipoprotein and lipid | mmol/L | 23568 | 23868 |
| Cholesterol in Medium LDL | Lipoprotein and lipid | mmol/L | 23540 | 23840 |
| Cholesterol in Medium VLDL | Lipoprotein and lipid | mmol/L | 23505 | 23805 |
| Cholesterol in Small HDL | Lipoprotein and lipid | mmol/L | 23575 | 23875 |
| Cholesterol in Small LDL | Lipoprotein and lipid | mmol/L | 23547 | 23847 |
| Cholesterol in Small VLDL | Lipoprotein and lipid | mmol/L | 23512 | 23812 |
| Cholesterol in Very Large HDL | Lipoprotein and lipid | mmol/L | 23554 | 23854 |
| Cholesterol in Very Large VLDL | Lipoprotein and lipid | mmol/L | 23491 | 23791 |
| Cholesterol in Very Small VLDL | Lipoprotein and lipid | mmol/L | 23519 | 23819 |
| Cholesteryl Esters in Chylomicrons and Extremely Large VLDL | Lipoprotein and lipid | mmol/L | 23485 | 23785 |
| Cholesteryl Esters in IDL | Lipoprotein and lipid | mmol/L | 23527 | 23827 |
| Cholesteryl Esters in Large HDL | Lipoprotein and lipid | mmol/L | 23562 | 23862 |
| Cholesteryl Esters in Large LDL | Lipoprotein and lipid | mmol/L | 23534 | 23834 |
| Cholesteryl Esters in Large VLDL | Lipoprotein and lipid | mmol/L | 23499 | 23799 |
| Cholesteryl Esters in Medium HDL | Lipoprotein and lipid | mmol/L | 23569 | 23869 |
| Cholesteryl Esters in Medium LDL | Lipoprotein and lipid | mmol/L | 23541 | 23841 |
| Cholesteryl Esters in Medium VLDL | Lipoprotein and lipid | mmol/L | 23506 | 23806 |
| Cholesteryl Esters in Small HDL | Lipoprotein and lipid | mmol/L | 23576 | 23876 |
| Cholesteryl Esters in Small LDL | Lipoprotein and lipid | mmol/L | 23548 | 23848 |
| Cholesteryl Esters in Small VLDL | Lipoprotein and lipid | mmol/L | 23513 | 23813 |
| Cholesteryl Esters in Very Large HDL | Lipoprotein and lipid | mmol/L | 23555 | 23855 |
| Cholesteryl Esters in Very Large VLDL | Lipoprotein and lipid | mmol/L | 23492 | 23792 |
| Cholesteryl Esters in Very Small VLDL | Lipoprotein and lipid | mmol/L | 23520 | 23820 |
| Concentration of Chylomicrons and Extremely Large VLDL Particles | Lipoprotein and lipid | mmol/L | 23481 | 23781 |
| Concentration of HDL Particles | Lipoprotein and lipid | mmol/L | 23430 | 23730 |
| Concentration of IDL Particles | Lipoprotein and lipid | mmol/L | 23523 | 23823 |
| Concentration of Large HDL Particles | Lipoprotein and lipid | mmol/L | 23558 | 23858 |
| Concentration of Large LDL Particles | Lipoprotein and lipid | mmol/L | 23530 | 23830 |
| Concentration of Large VLDL Particles | Lipoprotein and lipid | mmol/L | 23495 | 23795 |
| Concentration of LDL Particles | Lipoprotein and lipid | mmol/L | 23429 | 23729 |
| Concentration of Medium HDL Particles | Lipoprotein and lipid | mmol/L | 23565 | 23865 |
| Concentration of Medium LDL Particles | Lipoprotein and lipid | mmol/L | 23537 | 23837 |
| Concentration of Medium VLDL Particles | Lipoprotein and lipid | mmol/L | 23502 | 23802 |
| Concentration of Small HDL Particles | Lipoprotein and lipid | mmol/L | 23572 | 23872 |
| Concentration of Small LDL Particles | Lipoprotein and lipid | mmol/L | 23544 | 23844 |
| Concentration of Small VLDL Particles | Lipoprotein and lipid | mmol/L | 23509 | 23809 |
| Concentration of Very Large HDL Particles | Lipoprotein and lipid | mmol/L | 23551 | 23851 |
| Concentration of Very Large VLDL Particles | Lipoprotein and lipid | mmol/L | 23488 | 23788 |
| Concentration of Very Small VLDL Particles | Lipoprotein and lipid | mmol/L | 23516 | 23816 |
| Concentration of VLDL Particles | Lipoprotein and lipid | mmol/L | 23428 | 23728 |
| Free Cholesterol in Chylomicrons and Extremely Large VLDL | Lipoprotein and lipid | mmol/L | 23486 | 23786 |
| Free Cholesterol in IDL | Lipoprotein and lipid | mmol/L | 23528 | 23828 |
| Free Cholesterol in Large HDL | Lipoprotein and lipid | mmol/L | 23563 | 23863 |
| Free Cholesterol in Large LDL | Lipoprotein and lipid | mmol/L | 23535 | 23835 |
| Free Cholesterol in Large VLDL | Lipoprotein and lipid | mmol/L | 23500 | 23800 |
| Free Cholesterol in Medium HDL | Lipoprotein and lipid | mmol/L | 23570 | 23870 |
| Free Cholesterol in Medium LDL | Lipoprotein and lipid | mmol/L | 23542 | 23842 |
| Free Cholesterol in Medium VLDL | Lipoprotein and lipid | mmol/L | 23507 | 23807 |
| Free Cholesterol in Small HDL | Lipoprotein and lipid | mmol/L | 23577 | 23877 |
| Free Cholesterol in Small LDL | Lipoprotein and lipid | mmol/L | 23549 | 23849 |
| Free Cholesterol in Small VLDL | Lipoprotein and lipid | mmol/L | 23514 | 23814 |
| Free Cholesterol in Very Large HDL | Lipoprotein and lipid | mmol/L | 23556 | 23856 |
| Free Cholesterol in Very Large VLDL | Lipoprotein and lipid | mmol/L | 23493 | 23793 |
| Free Cholesterol in Very Small VLDL | Lipoprotein and lipid | mmol/L | 23521 | 23821 |
| Phosphatidylcholines | Lipoprotein and lipid | mmol/L | 23437 | 23737 |
| Phosphoglycerides | Lipoprotein and lipid | mmol/L | 23434 | 23734 |
| Phospholipids in Chylomicrons and Extremely Large VLDL | Lipoprotein and lipid | mmol/L | 23483 | 23783 |
| Phospholipids in HDL | Lipoprotein and lipid | mmol/L | 23414 | 23714 |
| Phospholipids in IDL | Lipoprotein and lipid | mmol/L | 23525 | 23825 |
| Phospholipids in Large HDL | Lipoprotein and lipid | mmol/L | 23560 | 23860 |
| Phospholipids in Large LDL | Lipoprotein and lipid | mmol/L | 23532 | 23832 |
| Phospholipids in Large VLDL | Lipoprotein and lipid | mmol/L | 23497 | 23797 |
| Phospholipids in LDL | Lipoprotein and lipid | mmol/L | 23413 | 23713 |
| Phospholipids in Medium HDL | Lipoprotein and lipid | mmol/L | 23567 | 23867 |
| Phospholipids in Medium LDL | Lipoprotein and lipid | mmol/L | 23539 | 23839 |
| Phospholipids in Medium VLDL | Lipoprotein and lipid | mmol/L | 23504 | 23804 |
| Phospholipids in Small HDL | Lipoprotein and lipid | mmol/L | 23574 | 23874 |
| Phospholipids in Small LDL | Lipoprotein and lipid | mmol/L | 23546 | 23846 |
| Phospholipids in Small VLDL | Lipoprotein and lipid | mmol/L | 23511 | 23811 |
| Phospholipids in Very Large HDL | Lipoprotein and lipid | mmol/L | 23553 | 23853 |
| Phospholipids in Very Large VLDL | Lipoprotein and lipid | mmol/L | 23490 | 23790 |
| Phospholipids in Very Small VLDL | Lipoprotein and lipid | mmol/L | 23518 | 23818 |
| Phospholipids in VLDL | Lipoprotein and lipid | mmol/L | 23412 | 23712 |
| Sphingomyelins | Lipoprotein and lipid | mmol/L | 23438 | 23738 |
| Total Cholines | Lipoprotein and lipid | mmol/L | 23436 | 23736 |
| Total Concentration of Lipoprotein Particles | Lipoprotein and lipid | mmol/L | 23427 | 23727 |
| Total Lipids in Chylomicrons and Extremely Large VLDL | Lipoprotein and lipid | mmol/L | 23482 | 23782 |
| Total Lipids in HDL | Lipoprotein and lipid | mmol/L | 23426 | 23726 |
| Total Lipids in IDL | Lipoprotein and lipid | mmol/L | 23524 | 23824 |
| Total Lipids in Large HDL | Lipoprotein and lipid | mmol/L | 23559 | 23859 |
| Total Lipids in Large LDL | Lipoprotein and lipid | mmol/L | 23531 | 23831 |
| Total Lipids in Large VLDL | Lipoprotein and lipid | mmol/L | 23496 | 23796 |
| Total Lipids in LDL | Lipoprotein and lipid | mmol/L | 23425 | 23725 |
| Total Lipids in Lipoprotein Particles | Lipoprotein and lipid | mmol/L | 23423 | 23723 |
| Total Lipids in Medium HDL | Lipoprotein and lipid | mmol/L | 23566 | 23866 |
| Total Lipids in Medium LDL | Lipoprotein and lipid | mmol/L | 23538 | 23838 |
| Total Lipids in Medium VLDL | Lipoprotein and lipid | mmol/L | 23503 | 23803 |
| Total Lipids in Small HDL | Lipoprotein and lipid | mmol/L | 23573 | 23873 |
| Total Lipids in Small LDL | Lipoprotein and lipid | mmol/L | 23545 | 23845 |
| Total Lipids in Small VLDL | Lipoprotein and lipid | mmol/L | 23510 | 23810 |
| Total Lipids in Very Large HDL | Lipoprotein and lipid | mmol/L | 23552 | 23852 |
| Total Lipids in Very Large VLDL | Lipoprotein and lipid | mmol/L | 23489 | 23789 |
| Total Lipids in Very Small VLDL | Lipoprotein and lipid | mmol/L | 23517 | 23817 |
| Total Lipids in VLDL | Lipoprotein and lipid | mmol/L | 23424 | 23724 |
| Total Phospholipids in Lipoprotein Particles | Lipoprotein and lipid | mmol/L | 23411 | 23711 |
| Total Triglycerides | Lipoprotein and lipid | mmol/L | 23407 | 23707 |
| Triglycerides in Chylomicrons and Extremely Large VLDL | Lipoprotein and lipid | mmol/L | 23487 | 23787 |
| Triglycerides in HDL | Lipoprotein and lipid | mmol/L | 23410 | 23710 |
| Triglycerides in IDL | Lipoprotein and lipid | mmol/L | 23529 | 23829 |
| Triglycerides in Large HDL | Lipoprotein and lipid | mmol/L | 23564 | 23864 |
| Triglycerides in Large LDL | Lipoprotein and lipid | mmol/L | 23536 | 23836 |
| Triglycerides in Large VLDL | Lipoprotein and lipid | mmol/L | 23501 | 23801 |
| Triglycerides in LDL | Lipoprotein and lipid | mmol/L | 23409 | 23709 |
| Triglycerides in Medium HDL | Lipoprotein and lipid | mmol/L | 23571 | 23871 |
| Triglycerides in Medium LDL | Lipoprotein and lipid | mmol/L | 23543 | 23843 |
| Triglycerides in Medium VLDL | Lipoprotein and lipid | mmol/L | 23508 | 23808 |
| Triglycerides in Small HDL | Lipoprotein and lipid | mmol/L | 23578 | 23878 |
| Triglycerides in Small LDL | Lipoprotein and lipid | mmol/L | 23550 | 23850 |
| Triglycerides in Small VLDL | Lipoprotein and lipid | mmol/L | 23515 | 23815 |
| Triglycerides in Very Large HDL | Lipoprotein and lipid | mmol/L | 23557 | 23857 |
| Triglycerides in Very Large VLDL | Lipoprotein and lipid | mmol/L | 23494 | 23794 |
| Triglycerides in Very Small VLDL | Lipoprotein and lipid | mmol/L | 23522 | 23822 |
| Triglycerides in VLDL | Lipoprotein and lipid | mmol/L | 23408 | 23708 |

**Supplementary Table 4. Drug classification and data coding.**

| **Drug Classification** | **Data Code** |
| --- | --- |
| Lipid-lowering medication | 20003, 6177, 6153 (1140861922; 1141146234; 1140910654; 1141192410; 1140861958; 1140888594; 1140888648; 1140861970; 1140910632; 140861946;1140862028; 1141192414; 1140861924; 1140861954; 1141157260; 1140861944; 1140861856; 1141157262; 1140861926; 1140861928; 1140861936; 1140861942; 140861858;1140888590;1140910652;1140861868;1140861892;1140861876; 1140861866; 1140861848; 1140861878;1140861894; 1140865576;1140909780; 1141157416) |
| Antihypertensive medication | 20003, 6177, 6153 (1140851692; 1140860696; 1140860706; 1140860714; 1140860728; 1140860736; 1140860750; 1140860752; 1140860758; 1140860764; 1140860776; 1140860784; 1140860790; 1140860802; 1140860806; 1140860878; 1140860882; 1140860892; 1140860904; 1140860912; 1140860918; 1140864618; 1140864910; 1140864952; 1140866078; 1140879802; 1140881706; 1140888552; 1140888556; 1140888560; 1140888646; 1140916356; 1140916362; 1140923718; 1141145660; 1141145668; 1141150328; 1141150560; 1141151016; 1141151018; 1141152998; 1141153006; 1141153316; 1141153328; 1141156836; 1141156846; 1141164148; 1141164154; 1141165470; 1141165476; 1141166006; 1141167758; 1141167822; 1141170544; 1141170870; 1141171336; 1141172492; 1141172682; 1141172686; 1141179974; 1141180592; 1141180598; 1141187788; 1141188408; 1141190934; 1141193282; 1141193346; 1141199940; 1141200698; 1141200726; 1141201038; 1141201040; 1140851332; 1140851336; 1140851338; 1140851362; 1140851364; 1140851368; 1140851660; 1140860332; 1140860404; 1140860422; 1140860562; 1140860738; 1140864950; 1140866072; 1140866074; 140866084; 1140866086; 1140866092; 1140866096; 1140866108; 1140866110; 1140866128; 1140866132; 1140866136; 1140866138; 1140866140; 1140866146; 1140866156; 1140866158; 1140866162; 1140866164; 1140866168; 1140866422; 1140866440; 1140866446; 1140888918; 1140888922; 1140909706; 1140916870; 1140917068; 1140926778; 1141146378; 1141180772; 1141180778; 1141188636; 1141194794; 1141194800; 1141194804; 1141194808; 1141194810; 1140851360; 1140851418; 1140851420; 1140851428; 1140851430; 1140851436; 1140860292; 1140860322; 1140860336;1140864550; 1140864574; 1140866220; 1140866226; 1140866232; 1140866236; 1140866244; 1140866248; 1140866280; 1140866282; 1140866306; 1140866308; 1140866312; 1140866318; 1140866324; 1140866328; 1140866330; 1140866332; 1140866334; 1140866340; 1140866352; 1140866354; 1140866356; 1140866360; 1140866388; 1140866390; 1140866396; 1140866400; 1140866402; 1140866404; 1140866406; 1140866410; 1140866416; 1140866418; 1140866420; 1140866426; 1140866438; 1140866442; 1140866444; 1140866448; 1140881894; 1140888512; 1140909708; 1140909722; 1140922324; 1140923272; 1140923276; 1140923282; 1140923402; 1140927174; 1140928624; 1141146128; 1141150898; 1141167108; 1141181520; 1141195254; 1141195258; 1141201250; 1140851508; 1140851522; 1140851556; 1140860172; 1140860180; 1140860232; 1140860250; 1140860266; 1140860278; 1140860308; 1140860314; 1140860318; 1140860320; 1140860324; 1140860328; 1140860330; 1140860338; 1140860340; 1140860342; 1140860348; 1140860356; 1140860358; 1140860386; 1140860394; 1140860396; 1140860398; 1140860402; 1140860406; 1140860410; 1140860426; 1140860434; 1140860492; 1140860498; 1140861090; 1140864176; 1140864410; 1140866704; 1140866712; 1140866724; 1140866738; 1140866756; 1140866758; 1140866764; 1140866766; 1140866778; 1140866782; 1140866784; 1140866798; 1140866800; 1140866802; 1140866804; 1140867734; 1140879758; 1140879760; 1140879762; 1140879818; 1140879824; 1140879834; 1140879854; 1140879866; 1140881702; 1140881722; 1140909368; 1140916628; 1140916730; 1140916868; 1140917076; 1140922930; 1140923336; 1141146124; 1141146126; 1141146184; 1141152076; 1141156754; 1141164280; 1141168498; 1141169516; 1141171152; 1141172742; 1141182904; 1141182968; 1141184324; 1141184722; 1141187048; 1141187780; 1140851730; 1140851784; 1140851786; 1140851790; 1140851800; 1140855976; 1140861088; 1140861106; 1140861110; 1140861114; 1140861120; 1140861128; 1140861130; 1140861136; 1140861138; 1140861176; 1140861190; 1140861202; 1140861276; 1140861282; 1140866460; 1140866466; 1140866484; 1140866546; 1140866554; 1140868036; 1140872472; 1140872568; 1140879810; 1140881692; 1140888510; 1140911088; 1140911698; 1140916930; 1140917428; 1140923572; 1140923618; 1140926188; 1140926780; 1140927934; 1140927940; 1140928212; 1140928226; 1140928234; 1141145870; 1141150500; 1141150538; 1141150926; 1141151474; 1141152600; 1141153026; 1141153032; 1141153394; 1141153454; 1141156656; 1141157136; 1141157140; 1141162546; 1141166752; 1141167832; 1141169096; 1141169710; 1141169730; 1141171804; 1141173766; 1141174684; 1141175224; 1141180238; 1141184390; 1141185444; 1141187056; 1141187094; 1141187774; 1141187962; 1141188152; 1141188576; 1141188936; 1141190548; 1141199858; 1141200400; 1141200782; 1141201814) |
| Antidiabetic medication | 20003, 6177, 6153 (1140857494; 1140874724; 1140857496; 1140874726;1140857500;1140874728; 1140857502; 1140874732; 1140857506; 1140874736; 1140874646; 1140874744; 1140874650; 1140874746; 1140874652; 1140883066; 1140874664; 1140884600; 1140874666; 1141152590; 1140874674; 1141153254; 1140874678; 1141153262; 1140874680; 1141156984; 1140874686; 1141171646; 1140874690; 1141171652; 1140874706; 1141177600; 1140874712; 1141177606; 1140874716; 1141189090; 1140874718; 1141189094) |

**Supplementary Table 5. Baseline characteristics of individual exposures among participants.**

| **Characteristics** | **Non-AAA** | **Incident AAA** | ***p*-Value** |
| --- | --- | --- | --- |
| N | 302811 | 1671 |  |
| Overall individual exposure burden score (SD) | 11.5±3.64 | 12.4±3.62 | < 0.001 |
| **Environmental pollution** | | | |
| NOx, μg/m^3^ (SD) | 43.3±15.2 | 43.2±15.3 | 0.799 |
| NO2, μg/m^3^ (SD) | 26.3±7.50 | 26.1±7.32 | 0.186 |
| PM2.5, μg/m^3^ (SD) | 9.9±1.03 | 9.9±1.02 | 0.826 |
| PM2.5-10, μg/m^3^ (SD) | 6.4±0.89 | 6.4±0.91 | 0.441 |
| PM10, μg/m^3^ (SD) | 16.2±1.88 | 16.2±1.91 | 0.971 |
| 24h noise, dB (SD) | 56.0±4.22 | 56.0±4.18 | 0.658 |
| Environmental pollution score (SD) | 3.0±2.05 | 3.0±2.02 | 0.818 |
| **Living environment** |  |  |  |
| Non-Greenspace percentage (SD) | 64.1±23.5 | 63.5±23.4 | 0.287 |
| Non-Bluespace percentage (SD) | 99.1±2.93 | 99.1±2.59 | 0.977 |
| Living environment score (SD) | 1.0±0.8 | 0.9±0.8 | 0.502 |
| **Socioeconomic** |  |  |  |
| Low household income (%) | 138221 (45.6%) | 1145 (68.5%) | < 0.001 |
| Unemployed (%) | 16996 (5.61%) | 117 (7.00%) | 0.016 |
| Low education attainment (%) | 194208 (64.1%) | 1336 (80.0%) | < 0.001 |
| Accommodation instability (%) | 144968 (47.9%) | 554 (33.2%) | < 0.001 |
| Index of Multiple Deprivation (SD) | 16.6±13.2 | 18.4±14.5 | < 0.001 |
| Socioeconomic score (SD) | 2.1±1.14 | 2.4±1.13 | < 0.001 |
| **Social psychology** |  |  |  |
| Lack of social support (%) | 107825 (35.6%) | 688 (41.2%) | < 0.001 |
| Socially inactive (%) | 86848 (28.7%) | 552 (33.0%) | < 0.001 |
| Social isolation (%) | 175670 (58.0%) | 912 (54.6%) | 0.005 |
| Living alone (%) | 53134 (17.5%) | 348 (20.8%) | < 0.001 |
| Loneliness (%) | 50899 (16.8%) | 276 (16.5%) | 0.775 |
| Emotional distress (%) | 133817 (44.2%) | 758 (45.4%) | 0.349 |
| Psychiatric diseases (%) | 25983 (8.58%) | 133 (7.96%) | 0.389 |
| Social psychology score (SD) | 2.1±1.30 | 2.2±1.38 | 0.003 |
| **Lifestyle** |  |  |  |
| Unhealthy diet (%) | 273720 (90.4%) | 1500 (89.8%) | 0.409 |
| Drinkers (%) | 159575 (52.7%) | 837 (50.1%) | 0.035 |
| Physically inactive (%) | 96570 (31.9%) | 622 (37.2%) | < 0.001 |
| Sedentary time (SD) | 3.7±2.11 | 4.5±2.28 | < 0.001 |
| Unhealthy sleep pattern (%) | 80379 (26.5%) | 551 (33.0%) | < 0.001 |
| Smoking status (%) |  |  | < 0.001 |
| Never | 120121 (39.7%) | 251 (15.0%) |  |
| Previous | 154340 (51.0%) | 943 (56.4%) |  |
| Current | 28350 (9.36%) | 477 (28.5%) |  |
| Lifestyle score (SD) | 3.3±1.18 | 3.8±1.09 | < 0.001 |

*p*-Values were determined using the ANOVA test for continuous variables and the chi-square test for categorical variables.

**Supplementary Table 6. Baseline characteristics of included and excluded participants.**

| **Characteristics** | **Included participants** | **Excluded participants** |
| --- | --- | --- |
| N | 304482 | 197668 |
| Age, years (SD) | 56.1±8.07 | 57.1±8.08 |
| Male (%) | 146208 (48.0%) | 74076 (41.6%) |
| White ethnicity or race (%) | 292232 (96.0%) | 165843 (93.1%) |
| BMI (SD) | 27.2±4.54 | 27.9±4.59 |
| **Medical history** | | |
| Hypertension (%) | 90423 (29.7%) | 65608 (33.2%) |
| Diabetes (%) | 22371 (7.35%) | 19572 (9.90%) |
| Dyslipidemia (%) | 115005 (37.8%) | 74008 (37.4%) |
| Chronic respiratory diseases (%) | 40130 (13.2%) | 28285 (14.3%) |
| Chronic kidney disease (%) | 1066 (0.35%) | 1021 (0.52%) |
| Cardiovascular disease (%) | 30989 (10.2%) | 24984 (12.6%) |
| Lipid-lowering medication (%) | 45046 (14.8%) | 34371 (17.4%) |
| Antihypertensive medication (%) | 60977 (20.0%) | 47754 (24.2%) |
| Antidiabetic medication (%) | 9805 (3.2%) | 9176 (4.6%) |

Values are presented as mean ± SD for continuous variables and n (%) for categorical variables. Included participants were those included in the final analytical cohort. Excluded participants were eligible baseline participants who were not included in the final analytical cohort because of incomplete exposure, covariate, NMR metabolomics, or follow-up information. Because excluded participants had incomplete data by definition, percentages were calculated using available data for each characteristic.

**Supplementary Table 7. Subgroup analyses of the associations between multidomain exposure scores and the risk of incident AAA.**

| **Variable** | **Male** | | **Female** | | **≥ 60** | | **< 60** | | **Normal BMI** | | **Abnormal BMI** | |
| --- | --- | --- | --- | --- | --- | --- | --- | --- | --- | --- | --- | --- |
|  | **HR (95%CI)** | **Adjusted (*p*-Value)^#^** | **HR (95%CI)** | **Adjusted (*p*-Value)** | **HR (95%CI)** | **Adjusted (*p*-Value)** | **HR (95%CI)** | **Adjusted (*p*-Value)** | **HR (95%CI)** | **Adjusted (*p*-Value)** | **HR (95%CI)** | **Adjusted (*p*-Value)** |
| **Environmental pollution score** | | |  |  |  |  |  |  |  |  |  |  |
| low | Reference | Reference | Reference | Reference | Reference | Reference | Reference | Reference | Reference | Reference | Reference | Reference |
| middle | 1.10 (0.96-1.24) | 0.811 | 0.84 (0.61-1.16) | 0.999 | 1.05 (0.92-1.20) | 0.999 | 1.04 (0.79-1.36) | 0.999 | 1.27 (0.97-1.66) | 0.429 | 1.02 (0.89-1.16) | 0.999 |
| high | 1.12 (0.99-1.27) | 0.323 | 1.24 (0.94-1.65) | 0.675 | 1.09 (0.96-1.24) | 0.839 | 1.23 (0.95-1.58) | 0.571 | 1.15 (0.88-1.51) | 0.999 | 1.15 (1.02-1.31) | 0.133 |
| Per SD increasement | 1.03 (1.00-1.06) | 0.137 | 1.05 (0.98-1.11) | 0.701 | 1.02 (0.99-1.05) | 0.613 | 1.05 (1.00-1.11) | 0.265 | 1.04 (0.99-1.10) | 0.651 | 1.03 (1.00-1.06) | 0.106 |
| **Living environment score** | |  |  |  |  |  |  |  |  |  |  |  |
| low | Reference | Reference | Reference | Reference | Reference | Reference | Reference | Reference | Reference | Reference | Reference | Reference |
| middle | 1.03 (0.90-1.16) | 0.999 | 1.04 (0.76-1.42) | 0.999 | 1.05 (0.92-1.19) | 0.999 | 0.93 (0.71-1.22) | 0.999 | 0.95 (0.72-1.25) | 0.999 | 1.05 (0.92-1.19) | 0.999 |
| high | 1.04 (0.91-1.18) | 0.999 | 1.21 (0.89-1.63) | 0.999 | 1.05 (0.92-1.20) | 0.999 | 1.08 (0.84-1.41) | 0.999 | 1.05 (0.80-1.38) | 0.999 | 1.07 (0.93-1.21) | 0.999 |
| Per SD increasement | 1.02 (0.95-1.09) | 0.999 | 1.09 (0.93-1.27) | 0.999 | 1.02 (0.95-1.09) | 0.999 | 1.05 (0.92-1.20) | 0.999 | 1.03 (0.89-1.18) | 0.999 | 1.03 (0.96-1.10) | 0.999 |
| **Socioeconomic score** | |  |  |  |  |  |  |  |  |  |  |  |
| low | Reference | Reference | Reference | Reference | Reference | Reference | Reference | Reference | Reference | Reference | Reference | Reference |
| middle | 1.40 (1.24-1.57) | < 0.001 | 1.35 (1.02-1.78) | 0.169 | 1.53 (1.36-1.73) | < 0.001 | 1.01 (0.77-1.33) | 0.999 | 1.72 (1.33-2.22) | < 0.001 | 1.33 (1.18-1.50) | < 0.001 |
| high | 1.99 (1.71-2.31) | < 0.001 | 1.80 (1.26-2.58) | 0.007 | 1.76 (1.49-2.07) | < 0.001 | 1.86 (1.42-2.42) | < 0.001 | 2.76 (1.99-3.81) | < 0.001 | 1.86 (1.60-2.17) | < 0.001 |
| Per SD increasement | 1.29 (1.23-1.35) | < 0.001 | 1.26 (1.12-1.42) | 0.001 | 1.27 (1.21-1.33) | < 0.001 | 1.25 (1.14-1.37) | < 0.001 | 1.45 (1.31-1.60) | < 0.001 | 1.26 (1.20-1.32) | < 0.001 |
| **Social psychology score** | |  |  |  |  |  |  |  |  |  |  |  |
| low | Reference | Reference | Reference | Reference | Reference | Reference | Reference | Reference | Reference | Reference | Reference | Reference |
| middle | 1.16 (1.04-1.30) | 0.055 | 1.26 (0.95-1.68) | 0.565 | 1.11 (0.99-1.25) | 0.357 | 1.26 (0.96-1.66) | 0.507 | 1.59 (1.22-2.07) | 0.003 | 1.11 (0.99-1.25) | 0.428 |
| high | 1.60 (1.37-1.88) | < 0.001 | 1.69 (1.18-2.42) | 0.021 | 1.30 (1.10-1.55) | 0.011 | 2.17 (1.59-2.96) | < 0.001 | 2.31 (1.65-3.24) | < 0.001 | 1.51 (1.29-1.78) | < 0.001 |
| Per SD increasement | 1.14 (1.10-1.19) | < 0.001 | 1.15 (1.05-1.26) | 0.009 | 1.08 (1.04-1.13) | 0.001 | 1.24 (1.15-1.34) | < 0.001 | 1.26 (1.16-1.37) | < 0.001 | 1.12 (1.08-1.17) | < 0.001 |
| **Lifestyle score** |  |  |  |  |  |  |  |  |  |  |  |  |
| low | Reference | Reference | Reference | Reference | Reference | Reference | Reference | Reference | Reference | Reference | Reference | Reference |
| middle | 1.43 (1.26-1.61) | < 0.001 | 1.52 (1.14-2.02) | 0.019 | 1.38 (1.22-1.56) | < 0.001 | 1.76 (1.36-2.27) | < 0.001 | 1.57 (1.21-2.04) | 0.003 | 1.40 (1.24-1.59) | < 0.001 |
| high | 1.71 (1.49-1.96) | < 0.001 | 1.78 (1.29-2.46) | 0.003 | 1.61 (1.40-1.85) | < 0.001 | 2.21 (1.67-2.91) | < 0.001 | 2.64 (1.99-3.50) | < 0.001 | 1.57 (1.37-1.81) | < 0.001 |
| Per SD increasement | 1.26 (1.20-1.32) | < 0.001 | 1.26 (1.13-1.40) | < 0.001 | 1.23 (1.17-1.29) | < 0.001 | 1.35 (1.23-1.48) | < 0.001 | 1.44 (1.30-1.59) | < 0.001 | 1.22 (1.16-1.28) | < 0.001 |
| **Overall exposure score** | |  |  |  |  |  |  |  |  |  |  |  |
| low | Reference | Reference | Reference | Reference | Reference | Reference | Reference | Reference | Reference | Reference | Reference | Reference |
| middle | 1.38 (1.21-1.57) | < 0.001 | 1.01 (0.72-1.41) | 0.999 | 1.31 (1.14-1.49) | 0.001 | 1.31 (0.96-1.78) | 0.441 | 1.79 (1.34-2.38) | < 0.001 | 1.23 (1.07-1.41) | 0.015 |
| high | 1.74 (1.53-1.97) | < 0.001 | 1.74 (1.30-2.32) | 0.001 | 1.56 (1.37-1.78) | < 0.001 | 2.14 (1.64-2.81) | < 0.001 | 2.40 (1.82-3.16) | < 0.001 | 1.64 (1.44-1.86) | < 0.001 |
| Per SD increasement | 1.08 (1.06-1.09) | < 0.001 | 1.09 (1.05-1.13) | < 0.001 | 1.06 (1.05-1.08) | < 0.001 | 1.11 (1.07-1.14) | < 0.001 | 1.12 (1.09-1.16) | < 0.001 | 1.07 (1.06-1.09) | < 0.001 |

\#*p*-Values were Bonferroni adjusted. All analyses were adjusted for sex, age, self-reported race, BMI, baseline comorbidities (hypertension, diabetes, dyslipidemia, chronic respiratory disease, chronic kidney disease, and cardiovascular disease), and prior use of antihypertensive, antidiabetic, and lipid-lowering medications. In subgroup analyses, the stratifying variable was omitted from the adjustment set.

**Supplementary Table 8. Sensitivity analyses of the associations between multidomain exposure scores and the risk of incident AAA.**

| **Variable** | **Excluding events that occurred within the first two years of follow-up** | | **Competing risk regression model** | | **Excluding participants with a prior medical history** | |
| --- | --- | --- | --- | --- | --- | --- |
|  | **HR (95%CI)** | **Adjusted (*p*-Value)^#^** | **HR (95%CI)** | **Adjusted (*p*-Value)** | **HR (95%CI)** | **Adjusted (*p*-Value)** |
| **Environmental pollution score** | |  |  |  |  |  |
| low | 1 (Reference) | Reference | 1 (Reference) | Reference | 1 (Reference) | Reference |
| middle | 1.05 (0.93-1.19) | 0.999 | 1.05 (0.93-1.18) | 0.999 | 0.95 (0.67-1.35) | 0.999 |
| high | 1.14 (1.01-1.28) | 0.163 | 1.13 (1.01-1.27) | 0.386 | 1.16 (0.84-1.60) | 0.999 |
| Per SD increasement | 1.03 (1.01-1.06) | 0.066 | 1.03 (1.01-1.05) | 0.093 | 1.04 (0.97-1.11) | 0.999 |
| **Living environment score** | |  |  |  |  |  |
| low | 1 (Reference) | Reference | 1 (Reference) | Reference | 1 (Reference) | Reference |
| middle | 1.04 (0.92-1.17) | 0.999 | 1.03 (0.91-1.15) | 0.999 | 1.18 (0.84-1.66) | 0.999 |
| high | 1.09 (0.96-1.23) | 0.889 | 1.06 (0.94-1.19) | 0.999 | 1.11 (0.79-1.57) | 0.999 |
| Per SD increasement | 1.04 (0.98-1.11) | 0.999 | 1.02 (0.97-1.09) | 0.999 | 1.05 (0.89-1.25) | 0.999 |
| **Socioeconomic score** |  |  |  |  |  |  |
| low | 1 (Reference) | Reference | 1 (Reference) | Reference | 1 (Reference) | Reference |
| middle | 1.38 (1.24-1.55) | < 0.001 | 1.37 (1.23-1.52) | < 0.001 | 1.25 (0.90-1.72) | 0.906 |
| high | 1.92 (1.66-2.21) | < 0.001 | 1.88 (1.64-2.16) | < 0.001 | 1.89 (1.21-2.97) | 0.027 |
| Per SD increasement | 1.28 (1.22-1.34) | < 0.001 | 1.27 (1.21-1.33) | < 0.001 | 1.19 (1.05-1.35) | 0.031 |
| **Social psychology score** |  |  |  |  |  |  |
| low | 1 (Reference) | Reference | 1 (Reference) | Reference | 1 (Reference) | Reference |
| middle | 1.15 (1.03-1.28) | 0.071 | 1.16 (1.04-1.29) | 0.084 | 1.44 (1.06-1.96) | 0.101 |
| high | 1.52 (1.30-1.76) | < 0.001 | 1.56 (1.35-1.80) | < 0.001 | 1.94 (1.26-3.00) | 0.014 |
| Per SD increasement | 1.12 (1.08-1.17) | < 0.001 | 1.13 (1.09-1.18) | < 0.001 | 1.23 (1.10-1.37) | 0.001 |
| **Lifestyle score** |  |  |  |  |  |  |
| low | 1 (Reference) | Reference | 1 (Reference) | Reference | 1 (Reference) | Reference |
| middle | 1.43 (1.27-1.60) | < 0.001 | 1.43 (1.28-1.60) | < 0.001 | 1.33 (0.97-1.83) | 0.364 |
| high | 1.69 (1.48-1.92) | < 0.001 | 1.68 (1.49-1.91) | < 0.001 | 1.72 (1.18-2.49) | 0.021 |
| Per SD increasement | 1.25 (1.20-1.31) | < 0.001 | 1.25 (1.20-1.30) | < 0.001 | 1.24 (1.10-1.40) | 0.002 |
| **Overall exposure score** |  |  |  |  |  |  |
| low | 1 (Reference) | Reference | 1 (Reference) | Reference | 1 (Reference) | Reference |
| middle | 1.31 (1.15-1.48) | < 0.001 | 1.31 (1.16-1.48) | < 0.001 | 1.41 (1.00-1.99) | 0.247 |
| high | 1.71 (1.52-1.92) | < 0.001 | 1.69 (1.50-1.89) | < 0.001 | 1.94 (1.40-2.70) | < 0.001 |
| Per SD increasement | 1.08 (1.06-1.09) | < 0.001 | 1.08 (1.06-1.09) | < 0.001 | 1.08 (1.04-1.13) | < 0.001 |

\#*p*-Values were Bonferroni adjusted. All analyses were adjusted for sex, age, self-reported race, BMI, baseline comorbidities (hypertension, diabetes, dyslipidemia, chronic respiratory disease, chronic kidney disease, and cardiovascular disease), and prior use of antihypertensive, antidiabetic, and lipid-lowering medications.

**Supplementary Table 9. Association between the metabolite score and the risk of incident AAA.**

| **Variable** | **Incident AAA*** | **HR (95% CI)** | ***p*-Value** | **Schoenfeld Test (*p*-Value)** |
| --- | --- | --- | --- | --- |
| **Metabolic Score** | |  |  | 0.881 |
| low | 0.163 | 1 (Reference) | Reference |  |
| middle | 0.311 | 1.64 (1.40-1.92) | < 0.001 |  |
| high | 0.635 | 3.48 (2.98-4.07) | < 0.001 |  |
| Per SD increasement | | 1.38 (1.35-1.41) | < 0.001 | 0.292 |

*****The incidence of AAA was expressed as cases per 1,000 person years. All analyses were adjusted for sex, age, self-reported race, BMI, baseline comorbidities (hypertension, diabetes, dyslipidemia, chronic respiratory disease, chronic kidney disease, and cardiovascular disease), and prior use of antihypertensive, antidiabetic, and lipid-lowering medications.

**Supplementary Table 10. Mediating effects of key metabolites in the association between the overall exposure score and the risk of incident AAA.**

| **Metabolite** | **ACME (beta, 95%CI)** | **Mediation proportion (95%CI)** | **Adjusted (*p*-Value)^#^** |
| --- | --- | --- | --- |
| Glycoprotein Acetyls | 0.005 (0.004 to 0.006) | 6.19% (4.97% to 8.22%) | < 0.001 |
| Degree of Unsaturation | 0.004 (0.003 to 0.005) | 4.49% (3.27% to 6.57%) | < 0.001 |
| Docosahexaenoic Acid | 0.002 (0.002 to 0.003) | 2.85% (1.97% to 4.00%) | < 0.001 |
| Omega 3 Fatty Acids | 0.002 (0.001 to 0.003) | 2.41% (1.19% to 3.51%) | < 0.001 |
| Triglycerides in Large LDL | 0.002 (0.001 to 0.002) | 2.27% (1.62% to 3.11%) | < 0.001 |
| Albumin | 0.002 (0.001 to 0.002) | 2.01% (1.30% to 2.74%) | < 0.001 |
| Cholesteryl Esters in Small HDL | -0.002 (-0.003 to -0.002) | -2.47% (-3.26% to -1.89%) | < 0.001 |
| Concentration of HDL Particles | -0.002 (-0.002 to -0.001) | -1.71% (-2.34% to -1.25%) | < 0.001 |
| Cholesteryl Esters in Very Small VLDL | -0.001 (-0.002 to -0.001) | -0.65% (-0.99% to -0.44%) | < 0.001 |
| Cholesterol in Very Small VLDL | -0.001 (-0.002 to -0.001) | -0.56% (-0.85% to -0.27%) | < 0.001 |
| Concentration of Medium HDL Particles | -0.001 (-0.002 to -0.001) | -0.46% (-1.42% to -0.31%) | < 0.001 |
| Cholesteryl Esters in Medium HDL | -0.001 (-0.002 to -0.001) | -0.45% (-0.70% to -0.29%) | < 0.001 |
| Cholesterol in Medium HDL | -0.001 (-0.002 to -0.001) | -0.44% (-1.30% to -0.26%) | < 0.001 |

\#*p*-Values were Bonferroni adjusted. Abbreviations: ACME, average causal mediation effect.

**Supplementary Figure**

**Supplementary Figure 1. Study flowchart.**

**Supplementary Figure 2. Incidence of AAA according to categories of multidomain exposure scores.**

**Supplementary Table 3. Associations between domain specific exposure scores and the NMR metabolomic profile.**

(A-C) Associations between domain specific exposure scores and NMR metabolomics. (D) Overlap of significant NMR metabolites identified in the two-stage analysis. All analyses were adjusted for sex, age, self-reported race, BMI, baseline comorbidities (hypertension, diabetes, dyslipidemia, chronic respiratory disease, chronic kidney disease, and cardiovascular disease), and prior use of antihypertensive, antidiabetic, and lipid-lowering medications.

**Supplementary Figure 4. Spearman correlation heatmap of metabolites included in the elastic net cox model.**

**Supplementary Figure 5. Restricted cubic spline analysis of the association between the metabolite score and the risk of incident AAA.**

All analyses were adjusted for sex, age, self-reported race, BMI, baseline comorbidities (hypertension, diabetes, dyslipidemia, chronic respiratory disease, chronic kidney disease, and cardiovascular disease), and prior use of antihypertensive, antidiabetic, and lipid-lowering medications.
